# Uterine Micro-Environment and Estrogen-Dependent Regulation of Osteopontin Expression in Mouse Blastocyst

**DOI:** 10.3390/ijms140714504

**Published:** 2013-07-11

**Authors:** Qing-Zhen Xie, Qian-Rong Qi, Ying-Xian Chen, Wang-Ming Xu, Qian Liu, Jing Yang

**Affiliations:** 1 Center for Reproductive Medicine, Renmin Hospital of Wuhan University, Wuhan 430060, Hubei, China; E-Mails: qi_qianrong@126.com (Q.-R.Q.); chenyingxian_2007@126.com (Y.-X.C.); Wmxu609@msn.com (W.-M.X.); gabby0331@aliyun.com (Q.L.); dryangqing@hotmail.com (J.Y.); 2 Department of Obstetrics and Gynecology, Taihe Hospital, Shiyan 441000, Hubei, China

**Keywords:** implantation, estrogen, uterus, blastocyst, osteopontin

## Abstract

Embryo implantation is a highly synchronized bioprocess between an activated blastocyst and a receptive uterus. In mice, successful implantation relies on the dynamic interplay of estrogen and progesterone; however, the key mediators downstream of these hormones that act on blastocyst competency and endometrium receptivity acquisition are largely unknown. In this study, we showed that the expression of osteopontin (OPN) in mouse blastocysts is regulated by ovarian estrogen and uterine micro-environment. OPN mRNA is up-regulated in mouse blastocyst on day 4 of pregnancy, which is associated with ovarian estrogen secretion peak. Hormone treatment *in vivo* demonstrated that OPN expression in a blastocyst is regulated by estrogen through an estrogen receptor (ER). Our results of the delayed and activated implantation model showed that OPN expression is induced after estrogen injection. While estrogen treatment during embryo culture *in vitro* showed less effect on OPN expression, the tubal ligation model on day 3 of pregnancy confirmed that the regulation of estrogen on OPN expression in blastocyst might, through some specific cytokines, have existed in a uterine micro-environment. Collectively, our study presents that estrogen regulates OPN expression and it may play an important role during embryo implantation by activating blastocyst competence and facilitating the endometrium acceptable for active blastocyst.

## 1. Introduction

In mammals, embryo implantation occurs in a limited time known as the window of implantation, specifically, the time after ovulation and the full formation of the corpus luteum [1]. The maternal hormones from ovaries, including estrogen and progesterone are known as the principle hormones in this process [2]. According to the respective secretion level during the menstrual cycle or estrous cycle, it is widely believed that progesterone is essential for embryo implantation in almost all the mammals studied. The estrogen secreted during proliferative phase is mainly responsible for endometrial epithelium proliferation, whereas the function of estrogen secreted in the luteal phase was species-specific [3,4]. In species including monkey, rabbit and hamster, progesterone alone is sufficient for implantation, while it remains indistinct whether implantation requires ovarian estrogen in humans and primates [5,6]. In mice, the secretion of progesterone is stimulated by mating behavior and the formation of corpora luteum, and gradually increases during the progress of pregnancy, while the ovarian estrogen has two secreted peaks at the proestrous phase and pre-implantation phase on day 4 of pregnancy (day of vaginal plug as day 1). The delayed implantation model could be induced by ovariectomy on the morning of day 4 before the estrogen secretion peak, the embryo will enter into a quiescent state which could be maintained for few days under the condition of continuing progesterone treatment. Subsequently, if estrogen is appended, embryo implantation could be activated 24–48 h after progesterone priming [7]. In contrast, embryo implantation will occur appropriately if the ovariectomy is performed after the estrogen secretion peak [8]. Such a delayed-activated implantation model demonstrated that pre-implantation estrogen mainly interacts with its receptor α (ERα) and directs successful implantation in a progesterone primed endometrium. ERα expression peaks on day 5 of pregnancy suggest that ERα might regulate implantation [9]. Additionally, the finding that ER protein was localized in mouse blastocyst during implantation [10] indicates that estrogen may directly regulate the gene expression in blastocyst via binding to ERα.

In mammals, embryo implantation begins with apposition, attachment and adhesion between uterine luminal epithelium (LE) and conceptus trophoblast. Several studies have identified that blastocyst adhesion to and invasion into uterine endometrium require the existence of extra cellular matrix (ECM) which including fibronectin and OPN [11,12]. OPN, a member of ECM protein family, contains a Gly-Asp-Ser (RGD) sequence that binds to cell surface integrins to promote cell-cell attachment and cell spreading [13]. OPN protein is expressed at a high level in uterine epithelium during mid-secretory phase, decidual tissues and invading cytotrophoblst in humans [14,15], and the OPN/integrin αvβ3 complex has been identified as a marker of distinguishing receptive from non-receptive endometrium in clinical practice [16]. The study in pigs and sheep illustrated that the complexes of OPN and integrin subunits were constitutively expressed on trophectoderm and at the apical surface of uterine LE and uterine glandular epithelium (GE) during early pregnancy, to induce adhesion between LE and trophoblast and stimulate the cytoskeletal reorganization [17,18]. In mice, OPN mRNA was localized in uterine GE on day 4 and luminal epithelium on day 5 of pregnancy [19], as well as the immune cells surrounding the decidual cells during early pregnancy [20]. It is suggested that OPN plays an important role in mouse pregnancy, for the OPN-deficient mice display decreased pregnancy rate during mid-gestation without any abnormality in embryo number, suggesting peri-implantation pregnancy loss, and OPN expression in LE may provides a connection for conceptus to LE [21]. Chaen’s group [19] proposed that OPN secreted from uterine endometrial gland on day 4 is involved in adhesion complex assembly and responsible for blastocyst adhesion competence. However, Botquin’s group demonstrated the increase of OPN expression in mouse blastocyst compared to morula using *in situ* hybridization, in which the OPN mRNA is localized in inner call mass (ICM) on day 4 of pregnancy. However, the specific regulatory mechanism remains unknown [22].

Although it is well known that blastocyst activation and uterine receptivity establishment rely on ovarian estrogen secretion, the specific mechanism and concrete signal pathways by which estrogen facilitates blastocyst activation and adhesion to endometrium remain unknown. Here we demonstrated that OPN expression in mouse blastocyst is regulated by ovarian secreted estrogen together with uterine micro-environment. It is conceivable that estrogen may regulate blastocyst activation, adhesion, and uterine receptivity through up-regulating the mediator of OPN in blastocyst.

## 2. Results and Discussion

### 2.1. Results

#### 2.1.1. Increase of OPN Expression Corresponds to Ovarian Estrogen Surge

**Figure 1:**
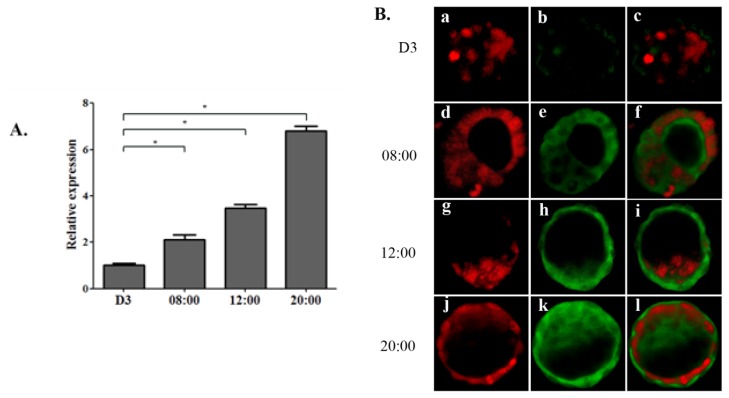
The expression and localization of osteopontin (OPN) in mouse blastocysts at the different phase of ovarian estrogen surge. (**A**) Real-time PCR detects the relative expression of OPN mRNA in mouse blastocysts collected from day 3 and day 4 at 08:00, 12:00 and 20:00 (*p < 0.05); (**B**) Representative embryonic immunofluorescence from day 3 (a–c) and day 4 at 08:00 (d–f), 12:00 (g–i) and 20:00 (j–l) by laser confocal microscopy, OPN and nuclei were stained with FITC (green) and PI (red), respectively.

OPN mRNA expression in mouse embryos on day 3 and 4 was quantitated by real-time PCR. Compared with the mRNA from the embryos collected on day 3, OPN mRNA expression was significantly up-regulated on day 4. OPN mRNA expression in blastocysts from 12:00 and 20:00 was much higher than that at 08:00, the time when estrogen surge occurs (Figure 1A).

In immunofluorescence protein localization experiments, increase of OPN protein expression was observed on day 4 periodically in mouse blastocysts, but not obvious on day 3 (Figure 1B(a–c)). The fluorescence intensity of blastocysts collected from 12:00 (Figure 1B(g–i)) and 20:00 (Figure 1B(j–l)) was greater than that from 08:00 (Figure 1B(d–f)). These findings demonstrated that OPN mRNA and protein are highly expressed in mouse blastocyst on day 4, specifically, the OPN expression pattern corresponds to the secretion pattern of ovarian estrogen.

#### 2.1.2. Ovarian Estrogen Is Necessary for Up-regulating OPN Expression in Mouse Blastocyst

**Figure 2:**
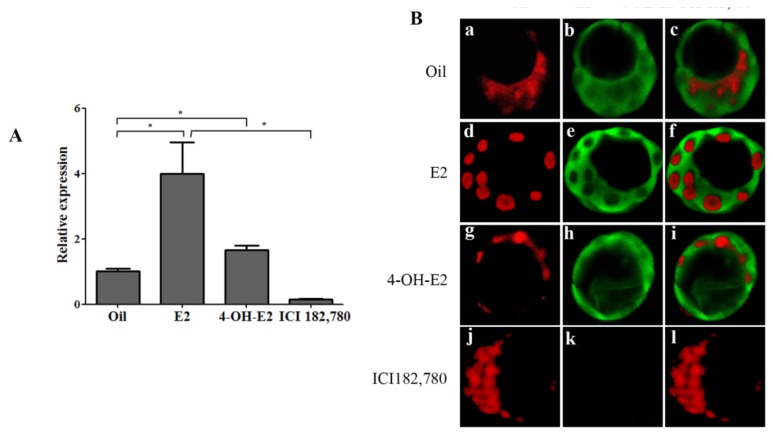
The expression and localization of OPN in mouse blastocysts from steroids treatment models. (**A**) Real-time PCR detects the relative expression of OPN mRNA in mouse blastocysts collected from steroids treatment models (* *p* < 0.05); (**B**) Representative embryonic immunofluorescence from steroids treatment models by laser confocal microscopy: oil injection group (a–c), E2 injection group (d–f), 4-OH-E2 injection group (g–i), ICI 182,780 injection group (j–l), OPN and nuclei were stained with FITC (green) and PI (red), respectively.

Because OPN was strongly expressed in blastocysts between and after ovarian estrogen secreted surge, steroids treatment was performed to examine whether OPN expression is regulated by estrogen *in vivo*. Mice were treated with estrogen (estradiol-17β, 17β-E2), 4-Hydroxyestradiol (estrogen metabolite, 4-OH-E2) and estrogen receptor α (ERα) antagonist (ICI 182,780) at 07:00 on day 4 by s.c. injection, respectively. Blastocysts were collected at 20:00 on day 4 for detecting the OPN expression. Our real-time PCR results showed that OPN mRNA expression is up-regulated by injecting estrogen at a dose exceeding physiological concentration (oil injection group). 4-OH-E2, an estrogen metabolite which could provide estrogenic activity, also increased OPN mRNA expression in mouse blastocysts. In contrast, ICI 182,780, an estrogen antagonist which could competitively bind to ERα, did not increase OPN mRNA level in blastocysts (Figure 2A).

To detect OPN protein expression in blastocysts, immunofluorescence experiment is performed. As shown in Figure 2B(d–f and g–i), the fluorescence intensity of blastocysts collected from estrogen (Figure 2B(d–f)) and 4-OH-E2 (Figure 2B(g–i)) treated groups was stronger than control group (Figure 2B(a–c)), while in the ICI 182,780 treated group, there was no fluorescence signal of OPN protein detected in blastocysts (Figure 2B(j–l)). These results from above indicated that ovarian estrogen regulates OPN expression, probably through ERα *in vivo*, and exogenous estrogen or estrogen metabolite were able to further up–regulate OPN expression.

**Figure 3:**
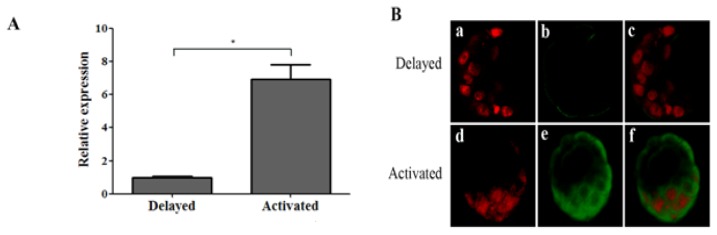
The expression and localization of OPN in mouse blastocysts from delayed and activated models. Ovariectomy was performed at 08:30–09:00 on day 4 of pregnancy, progesterone was injected to maintain the status of delayed implantation from days 5 to 7, dormant and activated blastocysts were collected before and 12 h after estrogen injection. (**A**) Real-time PCR detecting the relative expression of OPN mRNA in mouse blastocysts collected from delayed and activated model (* *p* < 0.05); (**B**) Representative embryonic immunofluorescence from delayed (a–c) and activated (d–f) model by laser confocal microscopy, OPN and nuclei were stained with FITC (green) and PI (red), respectively.

To further verify whether or not estrogen regulates OPN expression in blastocysts, the delayed and activated implantation model were utilized to detect OPN expression in dormant and activated blastocysts. Real-time PCR results showed that OPN mRNA expression is detected in activated blastocysts rather than dormant blastocysts (Figure 3A). Immunofluorescence results showed that the fluorescence intensity of activated blastocysts (Figure 3B(d–f)) is much higher than dormant blastocysts (Figure 3B(a–c)). Our delayed and activated implantation model supports the proposition that OPN expression in blastocysts needs the induction of estrogen.

#### 2.1.3. Ovarian Estrogen Induced OPN Expression Is Uterine Micro-Environment Dependent

**Figure 4:**
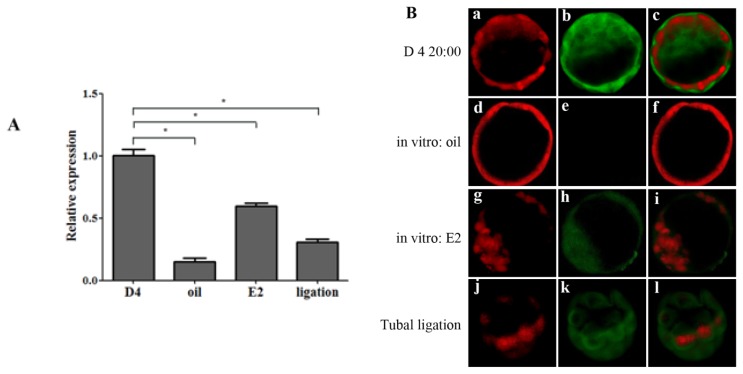
The expression and localization of OPN in mouse blastocysts from *in vitro* cultured and tubal ligation models. (**A**) Real-time PCR detects the relative expression of OPN mRNA in mouse blastocysts collected from *in vitro* cultured and tubal ligation model (* *p* < 0.05); (**B**) Representative embryonic immunofluorescence from *in vitro* cultured and tubal ligation model by laser confocal microscopy: the in vivo blastocysts on day 4 were taken as positive control (a–c), *in vitro* cultured with oil group (d–f), *in vitro* cultured with E2 group (g–i), tubal ligation (j–l), OPN and nuclei were stained with FITC (green) and PI (red), respectively.

To further study the regulation mechanism of OPN expression, morula collected from day 3 of pregnancy were cultured *in vitro*, estrogen was added in cultural medium to detect its influence on OPN expression in *in vitro* cultured blastocysts. Compared with the blastocysts collected from day 4 *in vivo*, the OPN mRNA expression was down-regulated in *in vitro* cultured blastocysts with or without exposed to estrogen (Figure 4A). These data led to a hypothesis that estrogen’s effect on OPN expression requires some factors or signal pathways existed in uterine micro-environment. To verify this hypothesis, we used tubal ligation model to examine whether the uterine micro-environment is essential for OPN expression in blastocysts *in vivo*. Under the condition of normal ovarian estrogen secretion, mouse oviducts were ligatured at 16:00 on day 3. Compared with blastocysts collected from day 4 at 20:00, OPN mRNA expression in blastocysts flushed from oviducts at the same time was down-regulated (Figure 4A), indicating that estrogen may regulate OPN expression in blastocyst indirectly which needs the uterine micro-environment. OPN protein expression in blastocysts from *in vitro* culture and tubal ligation model was examined by immunofluorescence (Figure 4B), which further support the quantitative results of mRNA expression level.

### 2.2. Discussion

Blastocyst activation is a committed step for successful implantation, by which blastocyst hatches from zona pellucida and acquires the competency of adhesion and invasion. At the same time, the uterine endometrium needs to transform to a spatiotemporally compatible environment where is favorable for blastocyst implantation [5]. Successful implantation begins with the process of apposition, adhesion, attachment and invasion between implantation-competent blastocysts and receptive uterus, and a group of adhesion molecules, including integrins, selectins, cadherin family [23–25]. Among them, integrin family are glycoprotein and multi-functional with ability to bind to RGD sequence which existed in extracellular ligands including fibronectin, OPN and others [26], to mediate cellular motility and adhesion. The integrin αvβ3, αvβ1 and α4β1 expressed during implantation window in humans and pigs could promote cell-cell attachment and spreading through bridging the bilateral ligand receptors on blastocysts and uterine epithelial cells [27].

OPN is a well-known ECM protein and cytokine contains RGD sequence, by which it could bind to integrin receptors expressed in cell surface and mediate cell adhesion, migration and differentiation [28]. It has been accepted that OPN-αvβ3 complex is a marker of uterine receptivity and implantation window [29], and large-scale transcriptomic approaches showed that OPN might participate in blastocysts-uterine epithelium interaction in humans and mice [16]. In pig, OPN mRNA is expressed by uterine LE and GE, but not by conceptus trophoblast, while OPN protein is present in uterine LE, GE and trophoblast, *in vitro* study illustrated that OPN binding to integrin receptors could induce cytoplasmic reorganization and focal adhesion in porcine uterine LE and conceptus trophoblast cell [18]. OPN is linked to the establishment and maintenance of pregnancy in ewes [30] and humans [31]. OPN mRNA is expressed in mouse blastocyst and specially localized in ICM on day 4 of pregnancy, but not the trophoblast [22]. In the mouse uterus, OPN mRNA is primarily localized in GE and weakly expressed in LE on day 4 [19]. Our quantitative results showed an increased level of OPN mRNA in mouse blastocyst compared to morula, while OPN proteins were localized in both ICM and trophoblast cells. As a secreted glycoprotein, OPN is existed in GE, LE and uterine lumen, indicating that the OPN protein found at trophoblast cells was of either uterine origin or ICM origin, and possibly involved in blastocyst-uterine interaction.

Previous studies suggested that progesterone and estrogen are the dominant controllers during embryo implantation [32,33]. In domestic ruminants, estrogen and progesterone secretion during peri-implantation period could induce the secretion of histotroph from GE to support embryo development and regulate the expression of molecules in LE to mediate embryo adhesion and implantation [34,35]. In mice, the activation of blastocyst and uterine receptivity will be induced by the cooperation of estrogen and progesterone on day 4 [36]. Among them, estrogen may play a key role in determining the duration of implantation window by the transient estrogen secretion surge on day 4 [37]. The blastocyst implantation occurs at the midnight of day 4. After that, gravid corpus luteum keeps on secreting progesterone which is required for decidualization, placentation and maintenance of pregnancy [38]. Estrogen secretion surge merged between 09:00 to 12:00 on day 4 [39], studies found that plentiful molecules expressed in uterine epithelium were estrogen-responsiveness, and have influence on the interaction between blastocyst and uterus [40], including leukemia inhibitory factor (LIF) [41], signal transducer and activator of transcription 3 (STAT3) [42].

Our results demonstrated that OPN mRNA and protein expression level was up-regulated on day 4 in mouse blastocysts, which is associated with ovarian estrogen secretion surge, suggesting that OPN is an estrogen-induced gene which might relate to adhesive and invasive capacity of blastocysts. Here we found that OPN expression in blastocysts was up-regulated at 12:00 and 20:00 compared to 08:00 before ovarian estrogen surge. Enhancing the OPN expression induced by exogenous estrogen and 4-OH-E2 indicates that OPN expressed in blastocysts is estrogen dependent. ICI 182,780 is an antagonist that competes with estrogen for ERα binding. The inhibition of OPN expression in mice injected with ICI 182,780 suggests that estrogen regulate blastocyst OPN expression via ERα. These data strongly argue that estrogen regulates OPN expression in mouse blastocysts. Delayed and activated implantation model provided a comprehensive description that estrogen is indispensable for blastocyst activation and uterine receptivity competency. The dormant blastocysts we collected before estrogen activation manifested as OPN expression inhibited, while OPN expression was up-regulated after estrogen treatment as expected. These results synthetically illustrated that OPN expression in blastocysts is closely associated with ovarian secreted estrogen on day 4.

In general, it has been thought that estrogen was not essential for decidualization and placentation in mice since mice performed with ovariectomy on day 5 of pregnancy are able to maintain pregnancy under the condition of progesterone supplement. However, Amrita’s group demonstrated that pregnant uterus could synthesize estrogen through de novo synthesis pathway, and decidualization may be compromised if uterine aromatase activity was inhibited [8]. In our study, OPN expression in blastocysts collected at 20:00 was higher than 12:00, part of reason might be that uterus synthesized estrogen has influence on OPN continuing expression. In order to studying the regulation manner of estrogen on OPN in blastocysts, we cultured morula till to blastocysts *in vitro*. Our experimental design is based on a hypothesis that estrogen could act directly on the embryos because of the presence of ER mRNA and protein in pre-implantation mouse embryos [10,43]. Beyond our expectation, compared to blastocyst formed *in vivo,* the OPN mRNA expression was down-regulated in *in vitro* cultured blastocysts, even with the supplementation of estrogen in culture medium. We deduced the possible reason for this might be that estrogen regulates OPN expression in blastocysts through the factors or signal pathways that exist in the uterus, rather than directly. We additionally established a tubal ligation model for supporting this conjecture. In our ligation model, the embryos were stagnated in oviducts on day 3, subsequently collected from oviducts at 20:00 on day 4, by which the blastocysts formed without uterine environment but with normal physiological estrogen *in vivo* were obtained. Quantification detection confirmed that OPN expression of blastocysts continuing existed in oviducts was restrained compared to “uterus checked-in” blastocysts *in vivo*. Taken together, excluding the canonical pathway that estrogen acts on blastocysts directly by binding to ER, estrogen is also likely to regulate the blastocyst competency indirectly via uterine micro-environment. However, the specific factors and signals involved need to be further investigated. These findings suggested that the regulation of blastocyst OPN expression by estrogen requires uterine micro-environment. However, the analysis of endometrial secretions contributing to peri-implantation events is too difficult for us to undertake noninvasively.

Recently, several studies focused on uterine secretomic analysis and found that some contents of secretions might play a pivotal role in embryo-endometrial cross-talk. The uterine LE and GE secrete proteins and nutrients into uterine lumen for embryo development, pregnancy recognition and implantation [44]. The micro-environment of uterine fluid which contains an abundance of nutrients, proteins, lipids and cytokines may participate in initiating implantation. Hannan’s work verified that vascular endothelial growth factor (VEGF), contained in endometrial secretions, could enhance blastocyst outgrowth and increase endometrial epithelia cell adhesion [45]. Clinical research found that leukemia inhibitory factor (LIF) level was decreased in the uterine flushing fluid of patients with adenomyosis [46]. Exosomes, the nanoparticles (30–150 nm in diameter) released from cells, can transfer small mRNAs and miRNAs to cells at distant sites through extracellular environment [47]. There were evidences proved that some exosomes, released from endometrial epithelium and contained specific miRNAs, could be transferred into blastocysts or epithelial cells to facilitate implantation [48]. OPN is secreted from uterine GE into uterine lumen immediately before and during embryo attachment, and OPN protein is present at the apical surfaces of both uterine LE and conceptus trophoblast. Studies found that OPN binds to intergrins expressed on LE and trophoblast cells *in vitro* to initiate integrin activation and outside-in signaling [17]. OPN is one histotroph in uterine flushings from humans and many domestic animals during early pregnancy [19,49]. Based on recent results, binding of OPN to integrin receptors on trophoblast cells not only stimulates adhesion and migration, but possibly has the effect on trophoblast proliferation [44]. Therefore, the localization of OPN protein in trophoblast cells may facilitate its proliferation, adhesive and invasive ability.

## 3. Experimental Section

### 3.1. Animal and Treatments

Sexually mature mice (Kunming White outbred strain, 6–8 weeks) maintained in a controlled environment (14 h light and 10 h dark cycle). All animal procedures were approved by the Institutional Animal Care and Use Committee of Wuhan University.

Female mice were super-ovulated by an injection of 7.5 IU of PMSG (Lizhu Company, Guangzhou, China) followed by 7.5 IU of hCG (Lizhu Company, Guangzhou, China) 48 h later. After hCG treatment, the mice were mated with fertile males of the same strain to induce normal pregnancy (the day of vaginal plug is the day 1 of pregnancy).

Embryo collection and culture: on days 3 and 4, pregnancy was confirmed by recovering embryos from oviducts or uteri, blastocysts were collected by flushing the uterine horn at the different stage of ovarian estrogen secretion surge (08:00, 12:00 and 20:00 on day 4) and prepared for the following experiments. For *in vitro* embryo culture, embryos at morula stage were collected by flushing the oviducts with PBS, and cultured in single step medium (Irvine Bio. Inc., Irvine, CA, USA) under mineral oil (Sigma-Aldrich Inc., St. Louis, MO, USA) at 37 °C, 5% CO_2_ for 24 h to blastocyst stage. For oviducts ligation treatment, mouse oviducts were ligatured by 6–0 silk thread at the joint part of uteri-oviduct under ether anesthesia at 16:00 on day 3 to make the embryos detained in oviducts, then the embryos were collected at 20:00 on day 4.

For steroids treatments, pregnant mice were treated with estradiol-17β (25 ng/mouse, Sigma-Aldrich Inc., St. Louis, MO, USA), 4-OH-E2 (50 ng/mouse, Sigma-Aldrich Inc., St. Louis, MO, USA) and ICI 182,780 (1 mg/mouse, AstraZeneca, London, United Kingdom) at 07:00 on day 4 by s.c. injection, respectively, pregnant mice were accepted sesame oil (Sigma-Aldrich Inc., St. Louis, MO, USA) as negative control. For *in vitro* embryo culture, estradiol-17β dissolved in ethanol was supplemented in medium, controls received ethanol only. For delayed and activated implantation model, pregnant mice were ovariectomized under ether anesthesia at 08:30–09:00 on day 4 of pregnancy. Delayed implantation was maintained by daily s.c. injection of progesterone (1 mg/mouse, Sigma-Aldrich Inc., St. Louis, MO, USA) on days 5–7. To terminate delayed implantation, progesterone-primed delayed-implantation mice were treated with estradiol-17β (25 ng/mouse, s.c.) on day 7. The blastocysts from delayed and activated mice were collected before or 12 h after estrogen treatment, respectively. For *in vivo* steroids treatment and model building, each group included five pregnant mice, for normal morula and blastocyst collection, as well as *in vitro* embryo culture, each group collected at least 100 embryos, and all the experiments will be repeated for three times.

### 3.2. RNA Extraction and Real-Time PCR

Mouse embryos were collected for total RNAs extraction using mRNA Capture Kit (Boehringer Mannheim, Germany) according to the manufacturer’s recommendations, total mRNA from blastocyst were reverse transcribed into cDNA by RNA PCR Kit (AMV) Ver.3.0 (TaKaRa Bio Inc, Tokyo, Japan).

For real-time PCR, cDNA was amplified using a SYBR Premix Ex Taq kit (TaKaRa Bio Inc, Tokyo, Japan) on the Rotor-Gene 3000A system (Corbett Research, Mortlake, Victoria, Australia). The conditions used for real-time PCR were as follows: 95 °C for 10 s, followed by 45 cycles of 95 °C for 5 s and 60 °C for 34 s. All reactions were run in triplicate. The corresponding primer sequences were used for real-time PCR. Gapdh served as internal control. Primer sequences used in real-time PCR for OPN were 5-CACTCCAATCGTCCCTAC-3 and 5-AGACTCACCGCTCTTCAT-3, for Gapdh were 5-GTTGTCTCCTGCGACTTCA-3 and 5-GGTGGTCCAGGGTTTCTTA-3. Data from real-time PCR were analyzed using the 2^−ΔΔ^*^Ct^* method. The significance of difference between two groups was assessed by Student’s *t*-test. The multiple comparison was performed with Tukey’s ANOVA. *p* < 0.05 was considered statistically significant.

### 3.3. Indirect Immunofluorescence

For indirect immunofluorescence, the mouse blastocysts were fixed in 4% paraformaldehyde solution for 30 min at 4 °C, washed in PBS 3 times, and treated with 1% triton X-100 (Sigma-Aldrich Inc., St. Louis, MO, USA) for 20 min at room temperature. After rinsed with PBS, the blastocysts were blocked with 5% bovine serum albumin (Sigma-Aldrich Inc., St. Louis, MO, USA) for 30 min at 37 °C, followed by incubation with mouse monoclonal OPN antibody (1:100 dilution, Santa Cruz Inc., Dallas, Texas, USA) or rabbit Ig G (1:100 dilution, Santa Cruz Inc., Dallas, Texas, USA) at 4 °C overnight. Then the blastocysts were incubated with FITC -conjugated Goat anti-Mouse Ig G (PIERCE, Rockford, IL, USA) for 1 h at 37 °C followed by three final two-minute PBS washes before being counter stained with PI (Sigma-Aldrich Inc., St. Louis, MO, USA), the signal was detected by laser confocal microscopy (LEICA, Solms, Germany).

## 4. Conclusions

In summary, our studies identified that the expression of OPN in mouse blastocyst is regulated by estrogen, partially through the cytokines or some other pathways existing in the uterine micro-environment. We supposed that OPN expression in blastocysts was, at least, partly involved in blastocyst activation to make them obtain the ability of adhesion and invasion, which is dependent on ovarian secreted estrogen. Although OPN is an estrogen responsiveness gene, the concrete regulation of estrogen on OPN expression is not direct but requires the uterine micro-environment. The possible pathway might be that estrogen activated some cytokines or OPN ligands through combining with its receptor, ERα. Since the OPN is associated with implantation competency of blastocysts, it is better to further study the specific regulatory mechanism on OPN expression.
